# *In vitro* experiment and computational fluid dynamics simulation study on blood pump for total cavopulmonary connection circulation

**DOI:** 10.3389/fbioe.2025.1564426

**Published:** 2025-04-01

**Authors:** Yong Wu, Tong Chen, Yunhan Cai, Shengzhang Wang, Haiyan Lu

**Affiliations:** ^1^ Center of Biotechnology and Biomedical Engineering, Yiwu Research Institute of Fudan University, Yiwu, Zhejiang, China; ^2^ Institute of Biomedical Engineering Technology, Academy for Engineering and Technology, Fudan University, Shanghai, China; ^3^ Institute of Biomechanics, Department of Aeronautics and Astronautics, Fudan University, Shanghai, China; ^4^ Department of Ultrasound, Shanghai East Hospital, Tongji University School of Medicine, Shanghai, China

**Keywords:** total cavopulmonary connection, right heart assist device, impeller, in vitro experiment, computational fluid dynamics simulation

## Abstract

**Objective:**

This study aimed to address the compromised hemodynamics in patients with total cavopulmonary connection circulation after Fontan surgery. While the Fontan procedure effectively separates systemic and pulmonary venous blood, resolving organ hypoxia, patients often experience complications such as elevated central venous pressure and reduced pulmonary artery pressure (Fontan failure) due to insufficient circulatory support. To improve this, a right ventricular assist device with a flexible impeller was designed. This study investigated the impeller’s characteristics through *in vitro* experiments and computational fluid dynamics (CFD) simulations, validating the accuracy and effectiveness of the CFD simulation method.

**Methods:**

The study employed *in vitro* hydraulic experiments and particle image velocimetry (PIV) to test the hydraulic performance and flow field of the blood pump. Simultaneously, a simulation model was established, and CFD simulations were performed. By quantitatively comparing simulation and experimental results, pulmonary artery blood flow, increased central venous pressure, and the velocity field in the mid-plane of the left pulmonary artery during impeller rotation were evaluated. The experimental setup was designed to mimic physiological dimensions, ensuring consistency with real-world applications.

**Results:**

The results demonstrated that the simulation method accurately predicted the trends of various indicators, with maximum errors within acceptable limits. Specifically, the relative error between simulation and experiment for pulmonary artery outflow was a maximum of 1.65%. The relative error for elevated central venous pressure was small, except for a few points. The simulation results of the velocity field also accurately reflected the main characteristics observed in the experiments.

**Conclusion:**

This study validated the potential of the designed impeller in improving hemodynamics in patients after Fontan surgery through *in vitro* experiments and CFD simulations. The high consistency between simulation results and experimental data confirms the effectiveness of the CFD simulation method, laying the foundation for further optimization of blood pump performance.

## 1 Introduction

Congenital heart disease with only one functional ventricle is collectively referred to as monoventricular heart disease. The average incidence rate of congenital heart disease is 9‰ (Botto et al., 2001). Single ventricular heart disease patients account for 9%–12% of all congenital heart disease populations (Pike et al., 2011). In a normal human body, systemic and pulmonary circulations are independent, and the arterial oxygen saturation is typically no less than 96% (Rao, 2021). However, in children with single ventricle heart disease, systemic and pulmonary blood flow mix within the single ventricle. The oxygen saturation of the blood in the systemic circulation is only 75%–85% (Rao and Vidyasagar, 2015) (depending on the ratio of pulmonary to systemic blood flow), leading to systemic organ hypoxia. Single ventricle heart disease primarily includes the following conditions: hypoplastic left heart syndrome, tricuspid atresia, double inlet left ventricle, unbalanced atrioventricular septal defect, and mitral atresia with a normal aortic root. To address this issue, surgical intervention is required to separate the systemic and pulmonary circulations, with the existing functional ventricle (whether morphologically a left or right ventricle) providing the circulatory pump function. Due to the high pulmonary artery pressure and resistance at birth, the surgery cannot be completed immediately and is divided into three stages. The classic three-stage surgical approach was proposed by De Leval et al. (1988). Taking the surgical process for tricuspid atresia as an example, the first stage surgery is typically performed during the neonatal period. Most patients have insufficient pulmonary perfusion, necessitating a Norwood procedure to increase pulmonary blood flow. This surgery connects the pulmonary artery to the aorta via a conduit or a stent, redirecting a portion of the aortic blood flow into the pulmonary artery, thereby aiding in the early development of the lungs. The second stage surgery, known as the Glenn procedure (Hopkins et al., 1985), is typically performed 3–6 months after birth. This procedure removes the shunt established in the first stage and then divides the superior vena cava (SVC). The proximal end of the SVC is ligated, and the distal end is connected to the right pulmonary artery via an end-to-side anastomosis, directing the blood flow from the upper body into the lungs and promoting further pulmonary development. The third stage surgery, known as the Fontan procedure, is performed between the ages of one and 2 years, with an interval of 1 year from the second stage surgery (Fontan and Baudet, 1971). This procedure directs the inferior vena cava (IVC) blood flow directly into the pulmonary artery, either by creating an intra-atrial tunnel or using an extracardiac conduit. The advantage of establishing an intra-atrial tunnel is an efficient and stable pathway, with less energy expenditure as blood flows through the tunnel (Puga et al., 1987). However, this method requires complex manipulations within the atrium, which can affect the sinoatrial node, potentially leading to arrhythmias, and the creation of an intra-atrial tunnel must be performed with the assistance of cardiopulmonary bypass (CPB). The advantage of using an extracardiac conduit is that it does not require CPB support and does not involve manipulation within the heart, thus having less impact on cardiac function. The disadvantages are that, compared to the intra-atrial tunnel, the extracardiac conduit requires blood to bypass the right heart, resulting in a longer conduit length and greater energy expenditure as blood flows through the conduit (Marcelletti et al., 1990). Currently, most surgeons opt for the extracardiac conduit approach to complete the Fontan procedure (Rao, 2021).

Following the three-stage surgical procedure, the patient’s SVC and IVC are directly connected to the pulmonary artery, establishing a total cavopulmonary connection (TCPC) structure. This bypasses the right heart, allowing venous blood to be directed into the lungs. The blood circulation formed by TCPC is called the Fontan circulation. Patients with single ventricular heart disease after undergoing Fontan surgery are called Fontan patients. While the Fontan procedure for single ventricular heart disease effectively separates arterial and venous blood, thereby addressing organ hypoxia, it does not fully resolve the issue of inadequate circulatory power. Patients with a single ventricle continue to depend on this single chamber for the circulation of blood to both the systemic and pulmonary systems. Over time, patients may exhibit signs of heightened central venous pressure and low pulmonary artery pressure (known as Fontan failure), which can lead to inadequate pulmonary perfusion, increased pulmonary vascular resistance, and systemic vascular resistance (Khairy et al., 2008). 40% of patients will experience early heart failure after Fontan surgery, and Fontan patients urgently need additional circulatory power to provide assistance (Throckmorton and Chopski, 2008). After decades of research, the use of mechanical devices to provide circulatory support for the human body has been proven safe and reliable, with the culmination of this research being ventricular assist devices (VADs). The earliest use of mechanical devices to provide circulatory support for patients with abnormal heart function can be traced back to the 1970s (Debakey, 1971). Based on the surgical approach, VADs can be classified into implantable VADs and percutaneous VADs. Implantable VADs require a surgical incision to open the chest, and the device is implanted within the body. Percutaneous VADs, on the other hand, do not require a surgical chest incision; the device itself, or its inlet and outlet cannulas, are introduced into the body via percutaneous interventional procedures.

Scholars have adopted existing ventricular assist devices or developed new devices to solve the problem of insufficient circulatory power in Fontan patients. Chopski et al. developed a transdermal axial blood pump in 2010 and placed it in the IVC of an ideal TCPC model to provide right heart assistance. The experimental results indicate that rapid rotation of the blood pump implanted in the IVC results in high-speed blood flow impacting the SVC flow, thereby hindering its entry into the pulmonary artery (Chopski et al., 2015). In 2013, Throckmorton et al. placed two pumps in the SVC and IVC respectively. It was found that the average retention time of blood in TCPC reduced to one-third of its original value, and the cell destructive factor increased by 60 times (Throckmorton et al., 2013). In 2014, Wang et al. implanted one end of an AvalonElite cannula with umbrella shaped polyurethane membrane into a TCPC animal model through the jugular vein, and the other end was connected to the cardiac pump CentriMag outside the body (Wang et al., 2014). Animal experiments have shown that this device can effectively improve the hemodynamic environment in single ventricle animals after Fontan failure, but thrombosis was found at the bottom of the umbrella shaped membrane. In 2019, Wang et al. further utilized artificial blood vessels with valves, AvalonElite double lumen cannula, and CentriMag centrifugal blood pumps to achieve double lumen assistance (Zhou et al., 2019). Animal experiments have shown that this device can effectively provide power assistance to both SVC and IVC. The commonality of the above auxiliary schemes is that although they can achieve good auxiliary effects, they require multiple devices to be used in combination, making them too cumbersome for clinical use.

**FIGURE 1:**
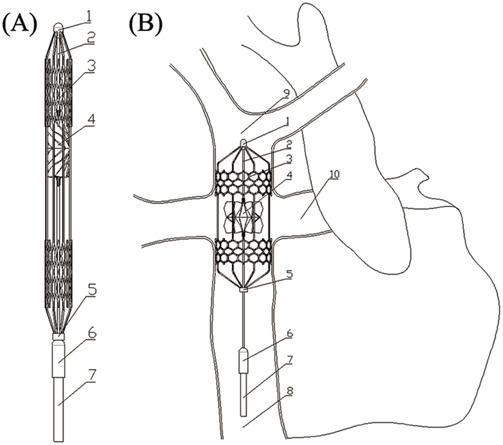
Overall structure of flexible double lumen assist device: **(A)** Compressed state; **(B)** Deployed state. (3-external stent; 4-impeller; 8-inferior vena cava; 9-superior vena cava; 10-pulmonary artery).

In order to compensate for the shortcomings of existing solutions, this paper proposes a right heart assist device with a flexible impeller to provide double lumen assistance for Fontan patients, as shown in Figure 1. The impeller and external stent of the device can undergo morphological changes. Before implantation, the overall device is in a contracted state, which is beneficial for reducing the trauma suffered by patients during surgery. After implantation, the device operates inside the TCPC. The impeller is a symmetrical structure that can provide power assistance for blood reflux from both SVC and IVC during rotation. And its hub has a diversion function, which can also avoid blood flow reversal and reduce single ventricular load when the impeller is stationary.

Through *in vitro* experiments and computational fluid dynamics (CFD) simulations, the characteristics of the blood pump impeller used for TCPC circulation were studied. The effectiveness and accuracy of the simulation method were verified by comparing various simulation results with experimental results, laying the foundation for further optimization of the blood pump impeller using CFD simulations.

## 2 Materials and methods

### 2.1 *In vitro* experimental apparatus

In order to maintain consistency with the actual application situation, an *in vitro* experimental device that conforms to physiological dimensions was designed. The *in vitro* experimental device consists of a bracket and a main body. The bracket is made of aluminum profiles and is divided into two parts, which are used to fix the tank and TCPC model respectively.

**FIGURE 2:**
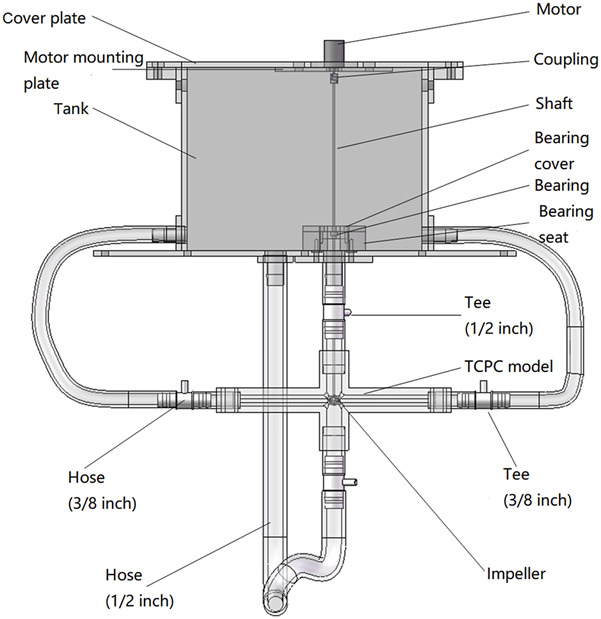
Main body of the *in vitro* experimental device.

The main body of the device is shown in Figure 2. During the experiment, the liquid is poured into the water tank from above, and the impeller rotates to drive the liquid to enter the TCPC from SVC and IVC respectively. Then, the liquid flows back into the water tank from the left pulmonary artery (LPA) and the right pulmonary artery (RPA) respectively, achieving the circulation of the liquid flow. The main components include a tank, cover plate, motor mounting plate, motor, coupling, shaft, bearing, bearing seat, TCPC model, impeller, 2 1/2-inch tees, 2 3/8-inch tees, 4 1/2-inch hoses, and 2 3/8-inch hoses. These components are divided into two categories: customized components and standard components.

The customized components within the main body of the *in vitro* experimental device include a water tank, bearing seat, bearing cover, cover plate, motor mounting plate, TCPC model, and impeller.

**FIGURE 3:**
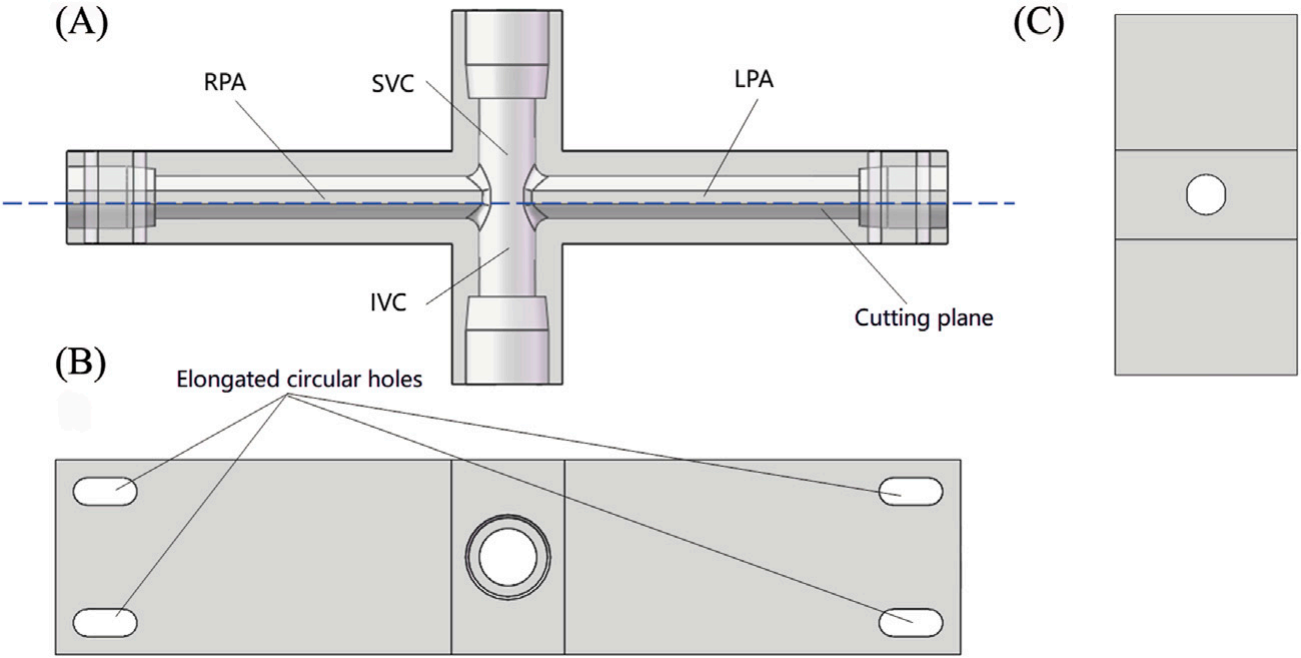
Schematic diagram of TCPC model: **(A)** Front view (perspective view); **(B)** Top view; **(C)** Side sectional view.

Figure 3 shows the specific structure of the TCPC model. The diameter of SVC and IVC is 12.7 mm, matching the standard inner diameter of 1/2-inch standard components. The cross-sections of LPA and RPA are shown in Figure 3C, with a diameter of 9.53 mm corresponding to the arc-shaped section, which matches the inner diameter of 3/8-inch standard components. For the convenience of camera shooting and laser incidence, the TCPC model is made entirely of transparent acrylic. In addition, the inner surface of the pipes needs to be carefully polished. For this purpose, the TCPC model is divided into two parts for processing. After the inner surface is polished, the two parts are glued together. The cutting plane of the model is represented by the red dashed line in Figure 3A. The fixation of the TCPC model is achieved through elongated circular holes at four corners, as shown in Figure 3B.

**FIGURE 4:**
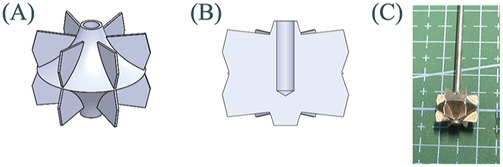
Schematic diagram of impeller: **(A)** Overall view; **(B)** Sectional view; **(C)** Processed rigid impeller.

Figures 4A,B show the overall structure and internal details of the impeller. To simplify experiments and simulations, the impeller is made of rigid materials. The diameter of the hub end is 2 mm, and the height of the impeller is 10 mm. The installation hole inside the impeller has a diameter of 1.5 mm and a depth of 6 mm. Figure 4C shows the processed rigid impeller made of SUS304 stainless steel. The average thickness of the leaves is 0.239 mm.

The standard components of the *in vitro* experimental device include a motor, DC power supply, shaft, coupling, bearing, tee, and hose. The motor is a hollow cup brushed motor 1723R from Jinyuan Electromechanical Company, with a rated voltage of 12 V, an unloaded speed of 10,700 rpm, and a locked rotor torque of 16.427 mNm. The considerations for selecting other standard components are as follows: when selecting a DC power supply, the main consideration is whether its output voltage range and current range meet the requirements. When selecting a shaft, the main consideration is whether its diameter matches the diameter of the impeller inner hole. When selecting a coupling, the main considerations are its inner hole diameter and the maximum torque it can withstand. When selecting bearings, the main considerations are their inner hole diameter and maximum speed. Tees and hoses have been determined according to the TCPC model design. Table 1 summarizes the information of all standard components.

**TABLE 1:** Summary of standard components.

Components	Manufactor	Model	Supplementary information
Motor	Jinyuan Electromechanical	1723R	Hollow cup brushed motor
Shaft	Yancheng Rongxing	304–1.5	Material:304 stainless steel Diameter:1.5 mm
Power Supply (Motor speedometer)	Hangzhou Fenle	FL1300A	Voltage range: 0–30 V Current range: 0–3 A Combining power supply and speed measurement functions
Coupling	Misumi	MCO6-1.5–1.5	Cross shaped coupling Fixed type with stop screw
Bearing	NSK	601XZZ	Inner diameter:1.5 mm, Outer diameter:6 mm Thickness:2.5 mm
Tee	RAUMEDIC	3/8inch 1/2inch	_
Hose	RAUMEDIC	3/8inch 1/2inch	_

### 2.2 Experimental platform

Two experimental platforms were built by combining the above *in vitro* experimental device with different equipment: Particle Image Velocimetry (PIV) experimental platform and Hydraulic experimental platform. The objective of conducting a PIV experiment is to capture the detailed local velocity field within the rotating impeller. The objective of hydraulic experiments is to evaluate the flow rate and pressure rise generated by the impeller across a range of rotational speeds.

#### 2.2.1 PIV experimental platform

**FIGURE 5:**
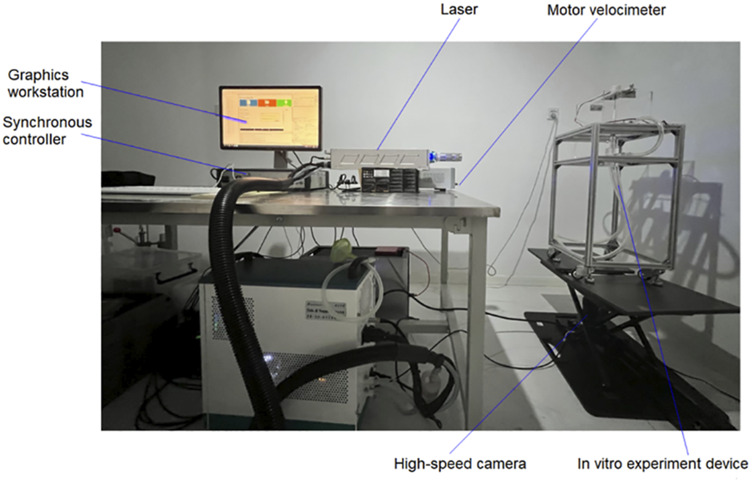
PIV experimental platform.

**FIGURE 6:**
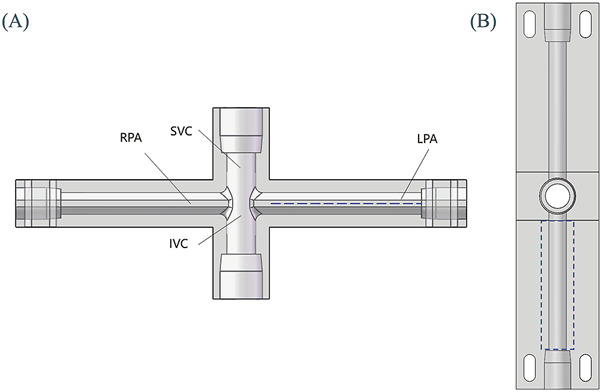
Camera’s shooting area in PIV experiment: **(A)** Front view; **(B)** Top view.

As shown in Figure 5, in addition to the *in vitro* experimental setup and motor velocimeter, it mainly includes a laser, tracer particles, high-speed camera, synchronous controller, and graphics workstation. The laser is a dual pulse Nd: YAG laser (Vlite-135, Dantec Dynamics, Denmark), consisting of a laser source and a laser emitter. The wavelength of the emitted laser is 532 nm, with an energy of 20 mJ. The tracer particles are PMMA-R aqueous suspension (micro particles GmbH) with a mass fraction of 5%, and the internal particle diameter is in the range of 1–20 µm. The high-speed camera is a CCD camera (FlowSenseEO4M, HiSense MKII, Danyec Dynamics) with a resolution of 2048 X 2048 pixels. Model of the synchronous controller is NC Pcle-427. Model of the graphics workstation is HP Z800, with built-in post-processing software Dynamic studio (Dantec Dynamics, Denmark). When conducting PIV experiments, the camera takes pictures from bottom to top, with the focus plane being the center plane of the LPA channel. Figure 6 shows the camera’s shooting area from a frontal and overhead perspective, respectively.

The fluid medium used in PIV experiments is determined based on its refractive index. The TCPC model is made entirely of transparent acrylic material. In order to ensure the accuracy of PIV experimental results, the refractive index of the internal medium should be consistent with that of the acrylic material. The refractive index of acrylic material is 1.491, while the refractive indices of benzyl alcohol and anhydrous ethanol are 1.539 and 1.361, respectively. The refractive index of a mixed solution was estimated based on the estimating formula (Huang et al., 2017). The optimal ratio of the mixed solution was further determined through experiments. Ultimately, PIV experiments were performed utilizing a blend of benzyl alcohol and anhydrous ethanol in a 20:7 volume ratio. The viscosity of the mixed solution was determined to be 0.0033 Pa·s using a viscometer.

#### 2.2.2 Hydraulic experimental platform

**FIGURE 7:**
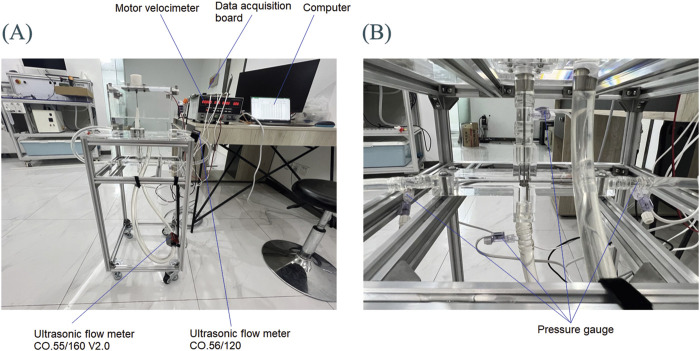
Hydraulic experimental platform: **(A)** Overall experimental platform; **(B)** Local situation of TCPC.

As shown in Figure 7, in addition to the *in vitro* experimental setup and motor speedometer, it also includes a flow meter, pressure gauge, data acquisition board, and a computer. Two ultrasonic flow meters (SONOTEC, Germany) were used to measure the flow inside each hose. The installation positions are shown in Figure 7A, and the models are CO.56/120 and CO.55/160 V2.0, respectively. During the experiment, the CO.56/120 flow meter was sequentially fixed to the LPA outlet and RPA outlet to measure the outlet flow rates of LPA and RPA. The CO.55/160 V2.0 flow meter was consistently mounted on the IVC inlet to accurately measure the incoming flow rates. Due to limited space, the flow meter could not be installed at the SVC inlet; thus, the SVC inlet flow rate was calculated using the principle of conservation of mass. Four customized pressure gauges (Biopigmal, Singapore) were used to measure the pressure at each inlet and outlet of TCPC, and their installation positions are shown in Figure 7B. The data acquisition board converted the physical signals collected by the flow meters and pressure gauges into electronic signals and transmitted them to the computer.

Using the measurement data from hydraulic experiments as boundary conditions for CFD simulation, we could obtain the local velocity field and compare it with the results of PIV experiments. For this purpose, the medium used in hydraulic experiments should be consistent with that used in PIV experiments. However, anhydrous ethanol used in PIV experiments had a corrosive effect on acrylic materials. Hence, a mixed solution of benzyl alcohol and anhydrous ethanol was not used in the hydraulic experiment, but a mixed solution of glycerol and water was used. To ensure the viscosity consistency required for the PIV experiment, the glycerol-to-water mass ratio was fine-tuned. Ultimately, a 4:6 glycerol-to-water mixture was chosen for hydraulic tests. The measured density was 1,104 kg/m^3^ and the measured viscosity was 0.0036 Pa·s. Both the density and viscosity were very close to the PIV experiment.

### 2.3 Simulation method

#### 2.3.1 CFD simulation model

**FIGURE 8:**
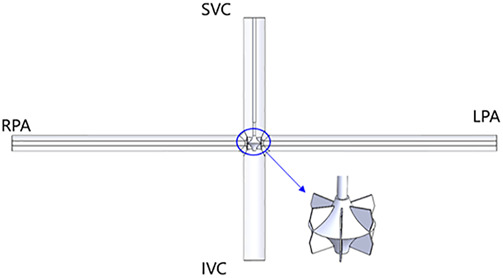
CFD simulation model.

A simulation model was created based on the experimental model 1:1, as shown in Figure 8. The influence of hollow circular tubes, shafts, and impellers on the flow field was considered. The distance from each entrance and exit to the center of the TCPC model is consistent with the distance from each pressure gauge in the experimental setup to the center of the TCPC. The distance from SVC and IVC inlet to TCPC center is 73 mm, and the distance from LPA and RPA outlet to TCPC center is 145 mm.

#### 2.3.2 CFD simulation settings

The creation of watershed grids was completed in Fluent Meshing 2020R2 (Canonsburg, United States), using polyhedral unstructured grids as the selected mesh type. In order to accurately capture the flow field near the wall, boundary layer grids were created at all wall locations. The boundary layer consisted of 10 layers of grids with a growth rate of 1.05. The height of the first layer grid was determined by an empirical formula $y^{+}$ (Zhihu, 2024). The determination of the primary grid size was conducted in accordance with established procedures for grid independence testing, ensuring that the simulation results are robust against variations in grid density. SVC and IVC inlets were set as flow boundary conditions, while LPA and RPA outlets were set as pressure boundary conditions. The flow and pressure data were both obtained from *in vitro* measurements. The material properties of the simulated medium were set to reflect those of a hydraulic experimental medium, with a density of 1,104 kg/m^3^ and a viscosity of 0.0036 Pa·s. The impeller surface and shaft surface were set as rotating surfaces, while the hollow circular tube surface and TCPC channel surfaces were set as stationary surfaces. The rotational surface speed and domain speed were set based on the measured speedometer data. The SST k−ω model was employed to simulate turbulent conditions. To ensure consistency with *in vitro* experiments, the CFD simulation also considered the influence of gravity, with the gravitational acceleration set at 9.8 m/s^2^. The examples were solved using a steady-state solver, with the convergence criterion set to a maximum residual of less than 10^−4^.

The boundary conditions for each entrance and exit in the TCPC model are shown in Table 2. With the gradual increase in speed, it is observed that the inlet flow rates of SVC and IVC, as well as the outlet pressures of LPA and RPA, all increase progressively, aligning with our expectations. At identical rotational speeds, the SVC’s inlet flow rate surpasses that of the IVC. This is because the fluid in the water tank travels a longer distance from IVC to the impeller than from SVC to the impeller. As the speed increases, the flow difference between the SVC inlet and IVC inlet gradually increases. At the same speed, the pressure at the LPA outlet and RPA outlet are very close. This is because TCPC is a left and right symmetric model, and the distance traveled by the fluid from the LPA outlet and RPA outlet back to the water tank is also approximately equal.

**TABLE 2:** Boundary conditions for CFD simulation of impeller.

Speed (rpm)	SVC flow rates (L/min)	IVC flow rates (L/min)	LPA pressure (Pa)	RPA pressure (Pa)
4,022	2.56	2.04	1890.2	1963.5
4,992	3.38	2.62	1993.5	2090.8
6,010	4.17	3.25	2129.8	2209.1
6,979	4.86	3.86	2303.1	2392.4
7,997	5.61	4.51	2555.7	2623.7

## 3 Results

### 3.1 Comparison of pulmonary arterial blood flow

Table 3 shows the simulation and experimental LPA and RPA outlet flow rates. The simulation results and experimental results show the same pattern: as the speed increases, the outlet flow rates of LPA and RPA gradually increase. At various speeds, the difference in outlet flow rates between LPA and RPA is relatively small, which is related to the left and right symmetry of the TCPC model and the approximately equal length of the hoses connecting the LPA and RPA outlets. The simulation results are very close to the experimental results, with a maximum relative error of only 1.65%, which demonstrates the reliability of the simulation method from the perspective of flow rate prediction.

**TABLE 3:** Comparison between simulation results and experimental results of left and right pulmonary artery outlet flow rates.

Speed (rpm)	LPA flow rates simulation (L/min)	LPA flow rates experiment (L/min)	Relative error (%)	RPA flow rates simulation (L/min)	RPA flow rates experiment (L/min)	Relative error (%)
4,022	2.30	2.32	0.86	2.30	2.28	0.88
4,992	3.03	3.04	0.33	2.95	2.96	0.34
6,010	3.73	3.78	1.32	3.70	3.64	1.65
6,979	4.44	4.46	0.45	4.28	4.26	0.47
7,997	5.22	5.20	0.38	4.90	4.92	0.41

### 3.2 Comparison of vena cava blood pressure rise

According to Table 3, the flow rate and pressure at the outlet of LPA and RPA are very close. Therefore, it can be assumed that fluid from the SVC and IVC inlets flows out equally from LPA and RPA. Furthermore, the mean outlet pressure of LPA and RPA can be used to subtract the inlet pressure of SVC and IVC, respectively, to obtain the blood pressure rise of the SVC and IVC (DP-SVC and DP-IVC).

**FIGURE 9:**
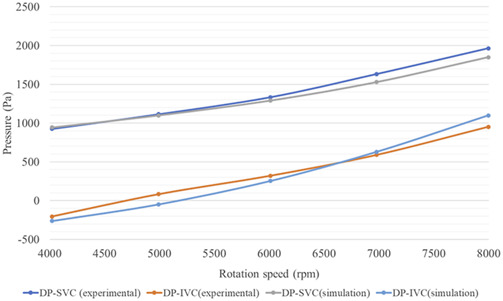
Comparison of simulation results and experimental results of blood pressure rise in SVC and IVC.

Comparative analysis of simulation and experimental data on blood pressure changes in the superior vena cava (SVC) and inferior vena cava (IVC) is illustrated in Figure 9, with detailed specific data and relative errors presented in Table 4. This approach aligns with methodologies used in cardiovascular hemodynamics research, such as those described in studies on the impact of myocardial bridging on coronary artery hemodynamics and numerical simulations of fluid-structure interactions in aneurysm treatment. According to Figure 9, it can be seen that the DP-SVC and DP-IVC obtained from simulation and experiment both continuously increase with the increase of speed, and the overall trend is consistent. When the speed increases from 4,022 rpm to 7,997 rpm, the simulated DP-SVC increases from 943.9 Pa to 1848.8 Pa, and the measured DP-SVC increases from 927.1 Pa to 1963.2 Pa. Based on this, it can be concluded that the average variation amplitude of DP-SVC obtained from simulation is smaller than that obtained from experiment. When the speed increases from 4,022 rpm to 7,997 rpm, the simulated DP-IVC increases from −262.1 Pa to 1099.4 Pa, and the measured DP-IVC increases from −204 Pa to 951.1 Pa. In contrast, the average variation amplitude of DP-IVC obtained from simulation is larger.

**TABLE 4:** Comparison between simulation results and experimental results of SVC and IVC blood pressure rise.

Speed (rpm)	DP-SVC simulation (Pa)	DP-SVC experiment (Pa)	Relative error (%)	DP-IVC simulation (Pa)	DP-IVC experiment (Pa)	Relative error (%)
4,022	943.9	927.1	1.82	−262.1	−204.0	24.49
4,992	1099.5	1115.1	1.40	−48.0	82.7	158.07
6,010	1290.4	1333.7	3.25	253.7	320.3	20.79
6,979	1528.3	1632.0	6.35	629.5	588.9	6.90
7,997	1848.8	1963.2	5.83	1099.4	951.1	15.60

According to the relative error data in Table 4, at 4,992 rpm, the simulated DP-IVC relative error is notably high, reaching 158.07%. This is due to the measured DP-IVC value being close to 0 at 4,992 rpm, resulting in a larger relative error due to the small absolute value. Further comparison between simulation and experimental results from the perspective of raw data is shown in Table 5. It is observed that the relative error between the simulated and experimental IVC inlet pressures is 6.67% at a speed of 4,992 rpm. The maximum relative errors of SVC and IVC inlet pressures are 18.26% and 9.05%, respectively, which are within an acceptable range. The above results demonstrate the reliability of the CFD simulation method for impellers from the perspective of pressure prediction.

**TABLE 5:** Comparison between simulation results and experimental results of SVC and IVC entrance pressure.

Speed (rpm)	SVC pressure simulation (Pa)	SVC pressure experiment (Pa)	Relative error (%)	IVC pressure simulation (Pa)	IVC pressure experiment (Pa)	Relative error (%)
4,022	983.0	999.8	1.68	2188.9	2130.8	2.73
4,992	942.7	927.1	1.68	2090.1	1959.5	6.67
6,010	879.1	835.8	5.18	1915.8	1849.2	3.60
6,979	819.5	715.8	14.48	1718.3	1758.9	2.31
7,997	740.9	626.5	18.26	1490.3	1638.6	9.05

### 3.3 Comparison of velocity field on the central plane

The raw images collected in PIV experiments need to be processed before they can be used for velocity field calculations. After determining the analysis area, the background is separated from the analysis area using segmentation algorithms. Then the background image is removed, leaving only the image of the analysis area for post-processing. Utilizing PIV technology, 200 sets of images were captured for the impeller at varying speeds, from which the corresponding velocity fields were derived. Then, MATLAB R2021b (MathWorks. Inc, Natick, Massachusetts, United States) is used to calculate the average velocity field, which is finally imported into PIV LAB to generate images. The velocity field of the same region is extracted from both simulation and experimental results for comparison.

**FIGURE 10:**
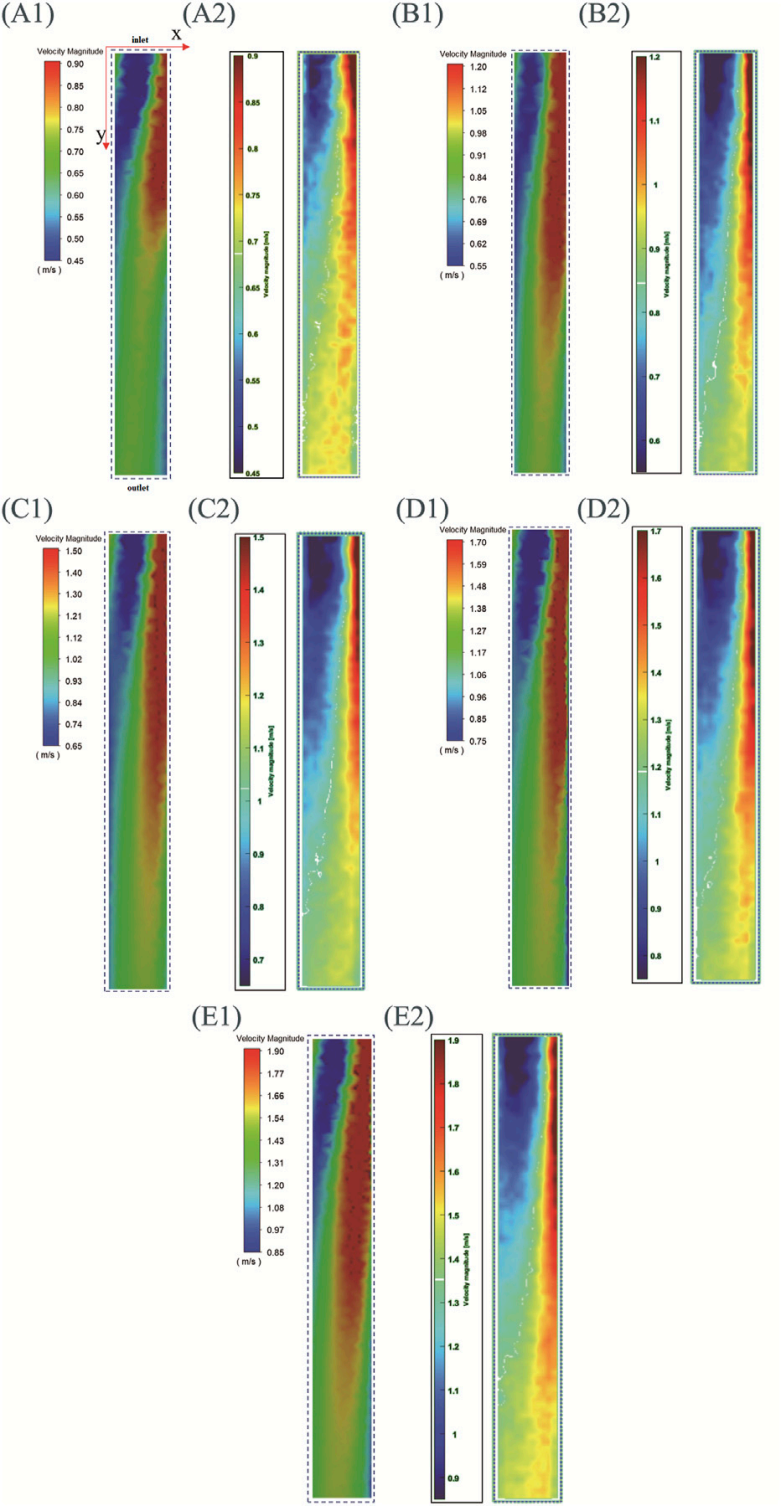
Simulation and experimental results of the central plane velocity field of LPA (with inlet connecting to SVC and IVC, outlet connecting to left lung). (A)–(E) speeds are 4,022 rpm, 4,992 rpm, 6,010 rpm, 6,979 rpm, and 7,997 rpm, **(A1**–**E1)** are simulation results, **(A2–E2)** are experimental results.

Figure 10 shows the simulation and experimental results of the velocity field. In order to accurately identify the characteristics of each velocity field, the velocity field corresponding to different rotational speeds is displayed with its respective optimal velocity ranges. When the rotational speeds are 4,022 rpm, 4,992 rpm, 6,010 rpm, 6,979 rpm, and 7,997 rpm, the minimum displayed speed of the velocity field is set to 0.45 m/s, 0.55 m/s, 0.65 m/s, 0.75 m/s, and 0.85 m/s respectively, and the maximum displayed speed is set to 0.9 m/s, 1.2 m/s, 1.5 m/s, 1.7 m/s, and 1.9 m/s respectively.

For the convenience of description, a two-dimensional coordinate system is defined: the coordinate origin corresponds to the upper left corner of the image, the positive direction of the x-axis corresponds to the direction from left to right, and the positive direction of the y-axis corresponds to the direction from top to bottom. The positive y-axis direction also represents the direction from the impeller to the outlet. Three regions are defined simultaneously: the blue region is the low-speed flow region, the red region is the high-speed flow region, and the region between blue and red is the medium-speed flow region. It can be seen that the experimental and simulation results corresponding to different rotational speeds exhibit the following characteristics: (1)As y increases, the velocity amplitude gradually decreases. This feature is consistent with the actual situation where the velocity of the fluid gradually decreases as it flows towards the outlet. (2)At the position close to the impeller (with a smaller y), the left side is the low-speed flow region and the right side is the high-speed flow region. As y increases (the fluid gradually moves away from the impeller), the area of high-speed flow region gradually increases and the area of low-speed flow region gradually decreases. This is because during the PIV experiment, images were captured by the camera from bottom to top, resulting in the velocity field displayed in Figure 10 reflecting this bottom-to-top observation. The rotation direction of the impeller is counterclockwise when viewed from bottom to top. So it can be concluded that under the driving effect of the impeller, the flow direction of high-speed fluid is also counterclockwise when leaving the impeller. Therefore, high-speed fluid will collide with the right pipe wall shown in Figure 10, resulting in a low-speed flow region on the left and a high-speed flow region on the right. After colliding with the pipe wall, the high-speed fluid moves towards the outlet while moving towards the left, resulting in an increase area of the high-speed flow region and a decrease area of the low-speed flow region.

There are also some differences between simulation results and experimental results: (1) The simulation results for each speed show a medium speed flow region in the top left corner of the image, absent in the experimental results. (2) At the same rotational speed, the simulation results show a larger high-speed flow region than the experimental results, and a smaller low-speed flow region than the experimental results.

In summary, although there are some differences between the simulation results and the experimental results in terms of areas of different regions, the simulation results accurately indicate the main characteristics of the velocity field. The above results demonstrate the reliability of the CFD simulation method for impellers from the perspective of velocity field prediction.

## 4 Discussion

### 4.1 Physiological conditions of SVC and IVC

Under normal physiological conditions, the IVC flow in the human body is greater than the SVC flow. However, according to Table 2, the IVC flow rate measured *in vitro* experiments is smaller than the SVC flow rate, which is related to the design of the experimental setup. According to Figure 7, it can be seen that the hose connected to IVC is longer than that connected to SVC, and the fluid in the water tank travels a longer distance through IVC to reach the impeller than through SVC. Therefore, the IVC flow rate corresponding to the rotation of the impeller is smaller than the SVC flow rate.

Analogizing the experimental setup to human circulation, fluid flows from the water tank through SVC to the impeller, corresponding to blood being pumped out of the left ventricle and circulating through the upper human body before returning to the right heart. Fluid flows from the water tank through IVC to the impeller, corresponding to blood flowing through the lower human body and returning to the right heart. The circulation distance of the lower body is indeed longer than that of the upper body, so the hose connected to IVC is longer than that connected to SVC, which is in line with physiological condition.

However, during the construction of the experimental setup, another physiological condition was not taken into account: the flow of blood in the lower body will receive certain assistance. During walking and movement, the contraction of calf muscles, often referred to as the ‘calf muscle pump’, compresses veins and, in conjunction with the anti-reflux function of venous valves, facilitates the upward flow of blood in the lower body. The experimental setup lacked components that mimic the calf muscles, rendering it incapable of simulating the assist effect of calf muscles. This ultimately led to results that the SVC flow rate was higher than the IVC flow rate during *in vitro* experiments.

### 4.2 Observation perspectives of PIV experiment

This article measures the velocity field on the central plane of LPA through PIV experiments and uses it as a reference to verify the accuracy of simulation results. The simulation outcomes exhibit a high degree of agreement with the experimental data, as evidenced by Figure 10. In addition to the plane mentioned above, the plane perpendicular to this plane is also the representative plane of the LPA. We omitted collecting velocity field information for another representative plane due to the following limitations: (1) Limitations of TCPC model processing technology. In order to ensure that the laser can smoothly irradiate the target area, the surfaces of each channel inside the TCPC model need to be precisely polished to ensure transparency. The current TCPC model is made by mechanical processing. If an integrated processing strategy is adopted, the polishing tool cannot penetrate deep into the interior of the model to polish the inner surfaces. Therefore, the TCPC model is divided into two parts for processing, and after the inner surfaces of the channels are polished, the two parts are then glued together. This will result in traces between the two parts. The current observation plane is parallel to the cutting plane, and the camera will not be affected by the cutting traces when shooting from bottom to top. To collect velocity information on another representative plane (the plane perpendicular to the current observation plane), the positions of the camera and the laser emitter need to be swapped, with the camera shooting from left to right and the laser shining from bottom to top. There will be obvious segmentation traces in the captured images of the camera, which will lead to serious discontinuity in the calculated velocity field. (2) Limitations of size and weight for laser emitters. Due to its relatively large size, placing the laser emitter beneath the *in vitro* experimental device necessitates elevating both the device and camera to a higher position. Maintaining the stability of these elevated *in vitro* experimental devices and cameras poses a considerable challenge. Furthermore, the laser emitter’s rear end, which is connected to the laser source via a pipeline, prevents it from being directly positioned on the bottom surface. How to ensure the heavy laser emitter in a vertical position will also be a challenge.

### 4.3 Limitations and prospects

A significant drawback of existing dual-lumen assist devices is that when the blood pump is stationary, it obstructs the inflow of blood from SVC and IVC into the pulmonary artery (Yang et al., 2023). Jagani et al. also demonstrated in their 2019 study that the energy expenditure for blood flow from SVC and IVC to the pulmonary artery without a device inside the TCPC was 9.344 mW, while this energy expenditure increased to 10.791 mW when a stationary dual-impeller device was present within the TCPC (Jagani et al., 2019). This is clearly contrary to the purpose of implantable assist devices. The impeller designed in this study has a unique flow-guiding structure that can effectively prevent blood flow collision from SVC and IVC, even when the impeller is stationary, guiding blood flow from the vena cavae to the pulmonary artery and reducing blood energy loss. However, comparative studies related to this are yet to be completed. In addition, this paper also has the following limitations: (1) During the design of the *in vitro* experimental device, the auxiliary impact of calf muscles on IVC blood reflux was overlooked. (2) The PIV experiment limited its data collection to velocity field information from a single plane. (3) Elastic deformation of the impeller was neglected in the design. (4) Patient-specific TCPC models were excluded from *in vitro* experimental processing. These will be improved in subsequent research through the following methods: (1) Through *in vitro* hydraulic experiments and CFD simulations, we will compare the impact of the proposed design in this study and existing assisting devices on venous return flow in a stationary state, quantitatively demonstrating the superiority of the proposed design. (2)Adding a device outside the hose connected to the IVC to simulate calf muscles thus to obtain blood flow distribution in SVC and IVC that is closer to physiological situation. (3) Using an integrated processing method that can ensure the roughness of the inner surface to construct TCPC models, and replacing the laser equipment with smaller size to collect more planar velocity field information. (4) Building a 3D PIV experimental platform (Chopski et al., 2015) to obtain three-dimensional flow field information inside TCPC to comprehensively verify the simulated flow field. (5) Using elastic materials to design flexible impellers and comparing the differences between rigid and flexible impellers during operation. (6) Using demolding technology (De Zélicourt et al., 2005) to create a patient specific TCPC model and conduct various *in vitro* experiments to study the performance of the impeller in the real TCPC structure.

## 5 Conclusion

This paper designs a blood pump for assisting TCPC circulation. Hydraulic performance tests and flow field tests of the blood pump were completed through *in vitro* experiments. Simultaneously, a 1:1 simulation model based on the experimental model was created for CFD simulations of the rigid impeller in rotation. The simulation results were quantitatively compared with the experimental results, including pressure increase in the vena cava blood flow, pulmonary artery blood flow rate, and velocity field in the central plane of the left pulmonary artery, when the impeller was rotating. The comparison results show that the simulation method used in this paper can accurately predict the trends of each indicator with the impeller speed, and the maximum error in each comparison is within an acceptable range. Therefore, the accuracy and effectiveness of the CFD simulation method used in this paper have been validated, and it can be used for further optimization of the blood pump’s performance.

## Data Availability

The original contributions presented in the study are included in the article/supplementary material, further inquiries can be directed to the corresponding authors.
